# Supplementary material for: Prevalence of Co-Infections with Respiratory Viruses in Individuals Investigated for SARS-CoV-2 in Ontario, Canada
Source: Viruses. 2021 Jan 18;13(1):130. doi: 10.3390/v13010130 (PMC7831481; doi:10.3390/v13010130)
Supplement: Supplementary file 1 [file viruses-13-00130-s001.pdf]

Figure S1.Diagram of specimens included in SARS-CoV-2 co-infection study
Study period: January 11 – April 20, 2020

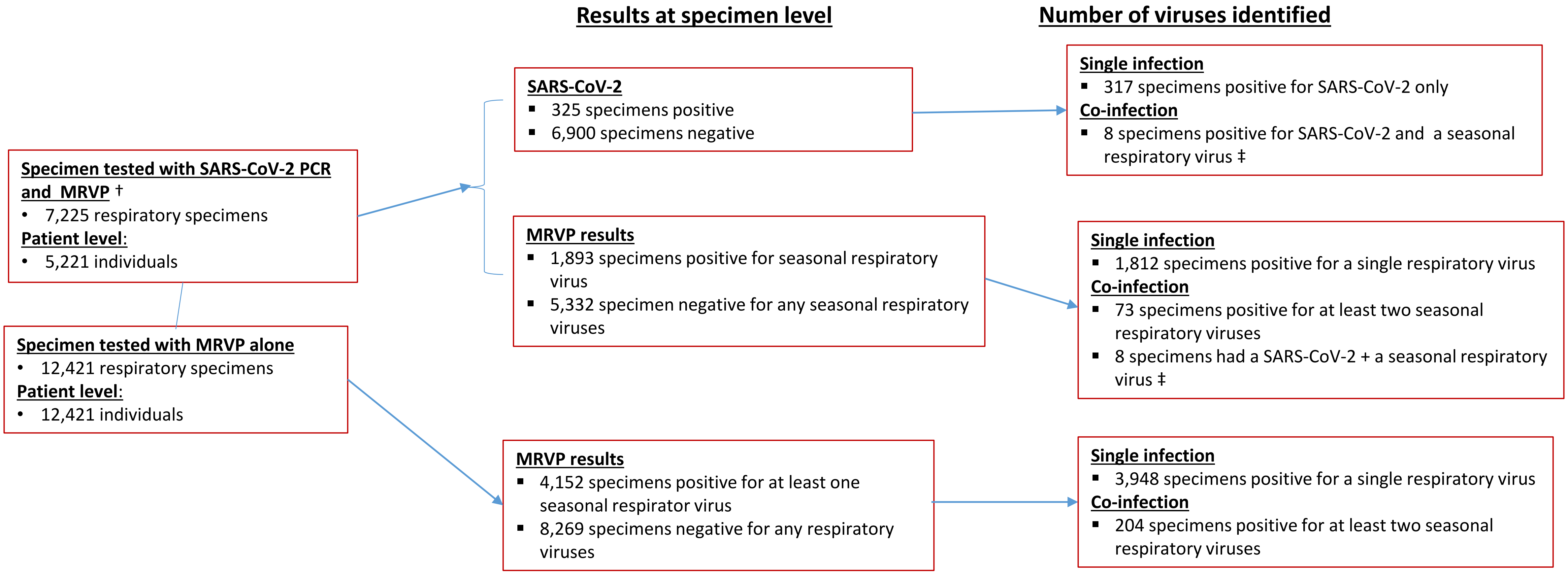

Footnote: † MRVP - Multiplex Respiratory Virus PCR assay;
‡ 8 specimens with SARS-CoV-2 co-infection identified represent the same specimens.
